# Feature interaction network based on hierarchical decoupled convolution for 3D medical image segmentation

**DOI:** 10.1371/journal.pone.0288658

**Published:** 2023-07-13

**Authors:** Longfeng Shen, Yingjie Zhang, Qiong Wang, Fenglan Qin, Dengdi Sun, Hai Min, Qianqian Meng, Chengzhen Xu, Wei Zhao, Xin Song

**Affiliations:** 1 Anhui Engineering Research Center for Intelligent Computing and Application on Cognitive Behavior (ICACB), College of Computer Science and Technology, Huaibei Normal University, Huaibei, Anhui, China; 2 Institute of Artificial Intelligence, Hefei Comprehensive National Science Center, Hefei, China; 3 Anhui Provincial Key Laboratory of Multimodal Cognitive Computing, School of Artificial Intelligence, Anhui University, Hefei, China; 4 School of Computer Science and Information Engineering, Hefei University of Technology, Hefei, Anhui, China; 5 Huaibei People’s Hospital, Huaibei, Anhui, China; 6 Anhui Big-Data Research Center on University Management, Huaibei, Anhui, China; University of Engineering & Technology, Taxila, PAKISTAN

## Abstract

Manual image segmentation consumes time. An automatic and accurate method to segment multimodal brain tumors using context information rich three-dimensional medical images that can be used for clinical treatment decisions and surgical planning is required. However, it is a challenge to use deep learning to achieve accurate segmentation of medical images due to the diversity of tumors and the complex boundary interactions between sub-regions while limited computing resources hinder the construction of efficient neural networks. We propose a feature fusion module based on a hierarchical decoupling convolution network and an attention mechanism to improve the performance of network segmentation. We replaced the skip connections of U-shaped networks with a feature fusion module to solve the category imbalance problem, thus contributing to the segmentation of more complicated medical images. We introduced a global attention mechanism to further integrate the features learned by the encoder and explore the context information. The proposed method was evaluated for enhance tumor, whole tumor, and tumor core, achieving Dice similarity coefficient metrics of 0.775, 0.900, and 0.827, respectively, on the BraTS 2019 dataset and 0.800, 0.902, and 0.841, respectively on the BraTS 2018 dataset. The results show that our proposed method is inherently general and is a powerful tool for brain tumor image studies. Our code is available at: https://github.com/WSake/Feature-interaction-network-based-on-Hierarchical-Decoupled-Convolution.

## Introduction

The purpose of medical image segmentation is to segment parts of medical images, extract related features, provide valuable information for quantitative evaluation of disease and formulation of treatment strategies, provide reliable assistance in pathological research and clinical diagnosis and deal with patient prognosis. Recently, deep learning has been widely used in the field of computer vision [1] and medical image processing.

**Fig 1:**
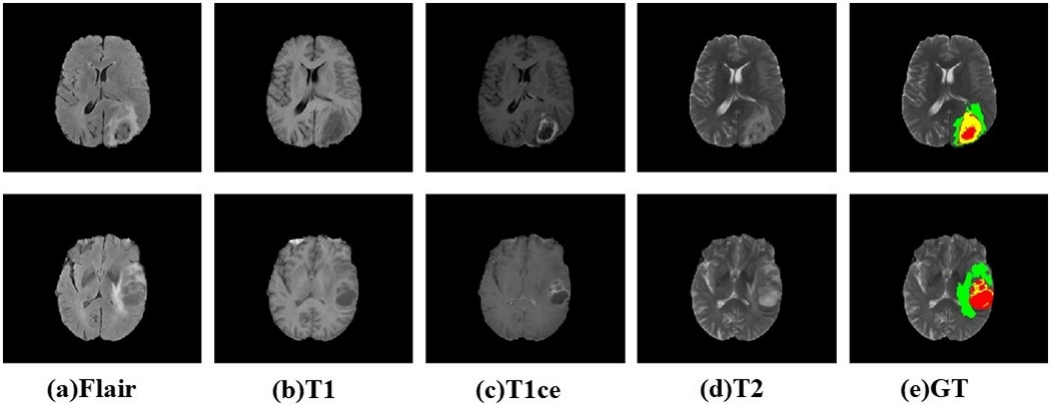
Two examples of multimodal image slices with ground truth from BraTS2018. In this figure, green represents GDEnhancing Tumor (numerical label 2), yellow represents Pertumoral Edema (numerical label 1), and red represents Necrotic and Non-Enhancing Tumor Core(NCR/ECT, numerical label 4) showing the differences in texture, size and shape of primary brain tumours.

Glioma [2] is a general term for tumors in the nervous system that originate from glial cells and neurons. As shown in Fig 1, it is the most common malignant tumor, accounting for 40%-50% of intracranial tumors. Glioma can be classified into astrocytoma, glioblastoma, oligodendroglioma, and so on, according to the type of glioma cells, which have different treatments and prognoses. Each type of glioma develops at a different age, with most younger patients having astrocytomas, middle-aged patients having glioblastoma multiforme, and children having myeloblastoma. These tumors have different shapes and sizes.

Accurate segmentation of brain tumors plays a vital role in early disease screening, evaluation of tumor progression and surgical treatment planning. However, the location and shape of lesions in different patients are quite different; experienced experts need to spend a lot of time and energy in marking the tumors manually. Automated segmentation methods can improve the efficiency of diagnosis and provide a visual representation of in vivo anatomy or function, which is essential for clinical analysis and medical intervention. However, this task faces the following challenges,

- The appearance, location, and shape of gliomas vary from patient to patient, making it difficult to accurately locate and segment smaller tumors.
- Brain tumors and normal tissues interpenetrate, making the borders blurred and indistinguishable.
- Imaging noise, low image contrast, unbalanced categories, and limited dataset sizes complicate this task.

Traditional segmentation methods of brain tumors are mainly based on random forest (RF) [3] classification, logistic regression (LR), and Markov random field (MRF) [4]. Most models are based on RF classifiers and the segmentation task is modeled as regularized stratified conditional random field (CRF), in which RF is used as a classifier. According to the local intensity information, each point in the brain is assigned a specific tissue category, and then these initial probability estimates are input into the radio frequency classifier together with the multimodal magnetic resonance imaging (MRI) to segment the brain tumor tissue. However, higher performance 3D convolutional neural networks (3D-CNNs) [5] emerged.

To improve the ability of deep convolutional neural networks in medical image segmentation, many attempts have been made. For example, the encoder-decoder structure has been improved to varying degrees, end-to-end learning [6] has been performed to maintain low-level features and obtain clear segmentation boundaries, atrous convolution [7] and multi-scale information effectively expands the receptive field, introduce attention learning mechanisms [8] in segmentation models, and make it possible to pay more attention to certain locations and channels. More novel methods add dual-stream pyramid module and context aware module [9] to the encoder decoder structure to avoid local feature loss. The attention mechanism is embedded in the convolution module to further refine the space and texture features [10], and make full use of the complementary advantages of three-dimension and two-dimensional convolution. For brain tumor segmentation, UNet [11] fully demonstrates the effectiveness of the U-shaped structure with outstanding results.

In terms of imaging methods, medical images are more diverse than natural images. However, medical images generally contain a large amount of noise due to the influence of imaging equipment, imaging principles, and individual differences. The preservation of image details must be taken into account while suppressing noise, which poses a great difficulty in lesion segmentation. Although some 2D CNN-based methods [12–14] have achieved impressive performance, most clinical imaging data is volumetric and these models ignore the critical 3D spatial information.

In 3D medical image segmentation tasks, 3D models [15–17] have demonstrated significant improvements over 2D models due to their ability to explore the contextual information contained in the slices, which is a great help in improving segmentation performance. However, compared to a conventional 2D CNN, the use of multi-layer 3D convolution encounters a higher computational cost due to the additional dimensionality. To solve this problem, some attempts have been made to reduce the number of network parameters that can be learned by using a lightweight network architecture [18, 19]. However, in terms of overall performance, these efficient models can not be compared with comprehensive models.

Therefore, re-visit the skip connection and attention of the U-shaped structure. UNet uses a simple skip connection to build a model with global multi-scale context information to achieve accurate segmentation of medical images, but a simple skip connection cannot effectively aggregate multi-scale features and the encoder cannot effectively mine enough information. For these reasons, it becomes a key problem to learn important local features at multiple scales, obtain semantic dependencies, and fuse the features learned by the encoder and decoder. In this study, we redesigned the structure of the skip connection and introduced a context-guided attentive conditional random field (CGA-CRF) module to connect the functions between the encoder and decoder. We introduced the feature fusion module into the skip connection to solve the class imbalance problem and improve the segmentation of complex medical images. We also introduced the global attention mechanism (GAM) [20] to further integrate the features learned by the encoder and explore the local context. The GAM module can reduce information diffusion and interact with features at the same time, which effectively solves the tumor variability problem.

The main contributions of this study are summarized as follows,

- Through simple analysis of the skip connection method, we find that the traditional simple connection method cannot realize the mutual learning between features.
- We propose a new approach by introducing a feature interaction module in the skip connection of the U-shaped network to enable information interaction and capture more accurate semantic information.
- We introduce a lightweight attention mechanism into the feature interaction structure of the U-shaped network for better feature learning and accurate segmentation of small tumors.

The structure of the rest of this article is as follows. The second section provides a review of relevant work, and the third section introduces our method in detail. The fourth part reports the data set descriptions, experimental results and performance analysis, followed by our conclusion in the fifth part.

## Related work

The application of deep neural networks in brain tumor segmentation has become a research focus for computer vision tasks because of its powerful automatic feature extraction and discrimination capabilities in supervised learning. In this section, we introduce recent methods related to glioma segmentation. Based on labeled and unlabeled training samples, existing glioma segmentation methods can be classified as supervised, semi-supervised, unsupervised, and hybrid learning, and supervised learning algorithms are the dominant approach. In the past few years, various deep neural network models for computer vision tasks have been proposed, such as ResNet [21] and DenseNet [22], which provide a new way to solve the MRI brain image segmentation problems and greatly contribute to the development of deep learning-based brain tumor diagnosis. Brain tumor segmentation methods based on unsupervised learning include threshold, region, active contour model, and clustering methods such as K-means clustering [23], Bayesian fuzzy clustering [24], fuzzy C-means clustering and superpixel clustering [25]. For supervised learning, early methods include support vector machines [26] and RF [27].

### Methods based on UNet

The traditional methods mentioned above require substantial manual interventions. Since 2015, UNet has adopted a symmetrical encoder architecture with skip connections, which gradually restores the down-sampled feature map to its original size, thus realizing the pixel-level intensive prediction of medical images. Later, UNet variants attracted a lot of attention and were further applied in medical image segmentation. UNet++ [28] reduces the semantic gap between encoder and decoder subnetworks by introducing a series of convolution dense connections and achieves better segmentation performance. 3D-UNet [29] replaces all 2D operations in UNet with 3D, such as 3D convolution, 3D pooling, and 3D up-sampling, which realizes better segmentation of medical image volume. RA-UNet [30] proposes a 3D hybrid residual attention perception segmentation method to precisely extract and segment tumors from the volume of interests, nnUNet [31] removes many of the excess bells and whistles from proposed network designs and focus on pre-processing and post-processing to achieve state-of-the-art performance in six recognized segmentation challenges. Probabilistic UNet [32] combines UNet with the conditional variational autoencoder (CVAE) to give UNet the ability to quantify prediction uncertainty. Partially reversible UNet [33] proposes a partially reversible UNet architecture that significantly reduces memory consumption and increases network depth to improve segmentation accuracy. 3D U2-Net [34] introduces depth-separable convolution to explore a promising general architecture. 3D dilated multi-fiber network [35] leverages the 3D multi-fiber units consisting of lightweight 3D convolutional networks to significantly reduce computational costs.

### Attention mechanism

In most classical models, such as UNet, the same low-level information is extracted consecutively at the beginning, which leads to redundant use of information. Attention mechanisms can be used to segment the features of the synapse area and suppress other noise parts [36]. To enhance the semantic information of the feature map, attention-UNet [37] introduces a channel attention mechanism based on UNet network, which compresses the features generated by UNet channel-by-channel, calculates the weight of the compressed features channel-by-channel, and then multiplies the weight with the original features to get the final features. GAU-Net [38] proposes a global attention mechanism, which integrates the channel attention module and the spatial attention module to obtain good segmentation performance. 3D attention UNet [39] adopts 3D UNet architecture and combines channel and spatial attentions with a decoder network to segment. SENet [40] proposes squeeze and excitation operations; squeeze operation obtains the global description characteristics, and excitation operation captures the relationship between channels. To improve the sensitivity of the model to channel characteristics, non-local neural networks [41] use non-local operations as simple, efficient, and general components to capture long-distance dependence in deep neural networks and solve the core problems of deep neural networks.

### Different skip connections

Skip connections are widely used to improve the performance and convergence of deep neural networks. The skip connection mechanism was first proposed in UNet, aiming to bridge the semantic gap between the encoder and decoder, and has proved to be effective in recovering the fine-grained details of the target objects. A fully convolutional network(FCN) also uses a skip connection; however, the difference is that the skip connection of FCN is added at the element level, while that of UNet is realized by the splicing of channels. UNet 3+ [42] uses a full-scale skip connection and depth supervision to combine high-level semantics with low-level semantics of feature maps from different scales, thus improving accuracy. With MultiResUNet [43], the feature map obtained by the encoder cannot be directly connected in series with the feature map output by the decoder, and there is a gap between them, and some convolutional layers are added to the path of the skip connection. Liu et al. [44] mainly analyzes and discusses some limitations of skip connections, and analyzes some limitations of batch normalization. A strategy of adaptively adjusting the input scale by recursive skip connections and layer normalization is proposed, which improves the performance of the skip connection.

## Proposed method

**Fig 2:**
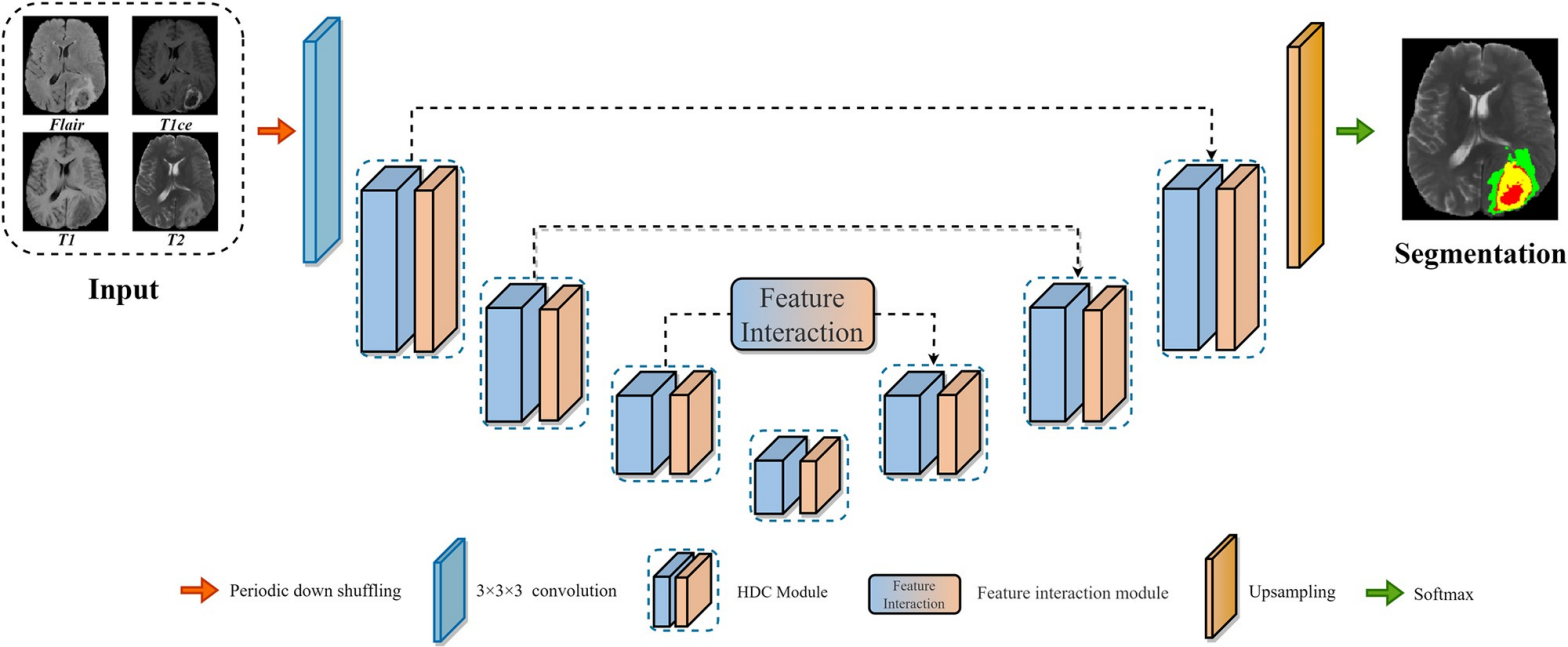
Diagram of the proposed method model, which has an encoder-decoder architecture.

In this section, starting with the lightweight hierarchical decoupled convolution(HDC) module [45], we detail a multi-modal brain tumor segmentation framework, as shown in Fig 2. This study combined the feature interaction module with the attention module and then extended HDCNet through the context-guided attentive CRF fusion module, to effectively integrate the context semantic features and the attention visual features.

### HDC module

Processing 3D medical images using deep structures, especially networks with complex self-attention, is often limited by large amounts of memory and computational power. Although the number of parameters can be greatly reduced using 2D convolution, they have inherent limitations in capturing rich spatial contexts. Within limited resource constraints, designing efficient kernels with low redundancy by decomposing standard convolutions, such as depth-separable convolutions [46], group convolutions [47], and decomposition convolutions is an effective way to address this problem.

**Fig 3:**
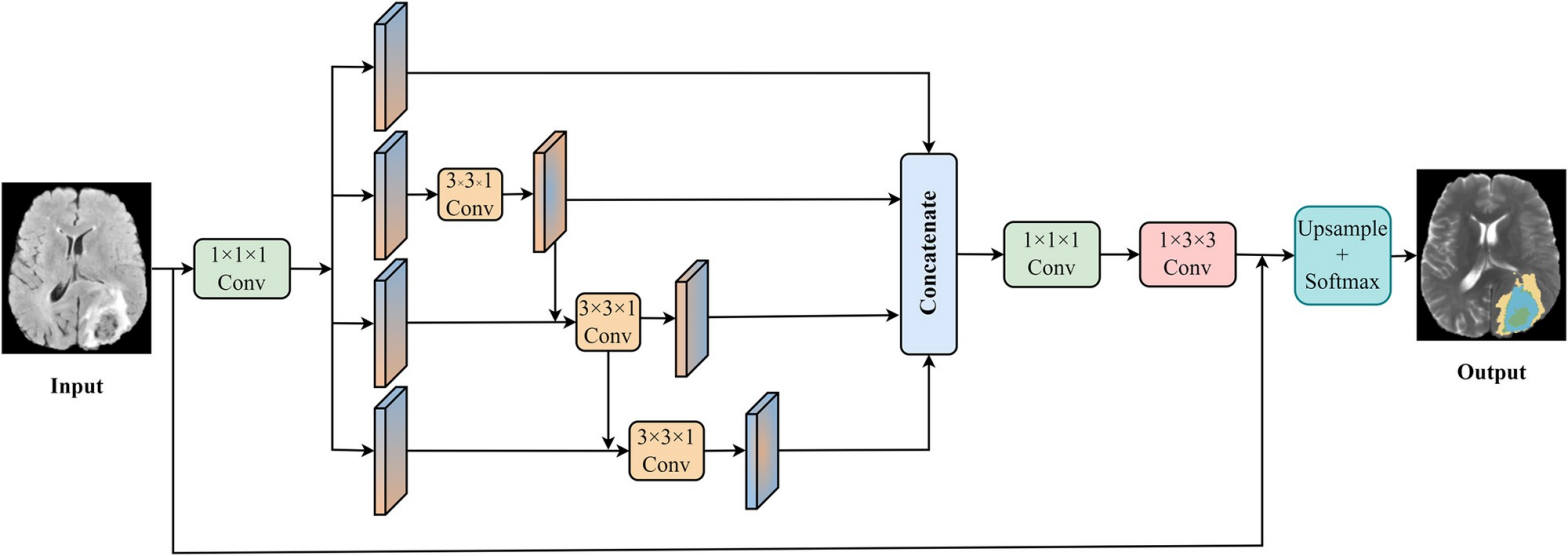
Hierarchical Decoupled Convolution(HDC) module.

Thus, Luo et al. [45] proposed a hierarchical decoupling convolution algorithm. As shown in Fig 3, the HDC module is not calculate simultaneously in space and channel dimensions like 3D convolution, but the standard convolution is decoupled along the space and channel dimensions. Based on the above method, to reduce the computational complexity and encode cues from multiple fields of view with minimal sacrifice of spatial context awareness, we use the HDC module to decompose the 3D spatial convolution in the spatial domain into two complementary 2D convolutions to introduce the view decoupled convolution. A new hierarchical group decoupling convolution is applied to the 2D convolution on the axis view of the channel domain, that is, the parallel axis view convolution is applied to the characteristic channel groups with a hierarchical connection. The main convolution applied to the parallel branches is used to extract the multi-scale features on the focused view of the 3D volume hierarchically, while the sub-convolution after the multi-branch module mixes the multi-scale output through the main convolution and extracts the spatial context features on the complementary view.

Experimental results show that, compared with the two-dimensional method, using an HDC module instead of a 3D convolution can extract more semantic features with a small amount of memory, and the hierarchical structure can make the network better use context information, thus obtaining more stable segmentation performance.

### Feature interaction module

Unbalanced categories and blurred boundaries are the difficult issues in medical brain tumor segmentation. In clinical diagnosis, experienced doctors usually determine the tumor boundary by the context information of its surrounding environment.

#### Projection with adaptive sampling

Adaptive sampling projection is a sampling-based image processing technology which is used to improve image quality. The pixels are sampled pixel by pixel, and the sampling density is adjusted adaptively according to the characteristics of the local image. When there are areas with high detail and complexity in the image, the resolution of these areas can be improved by increasing the sampling density, so that the image is clearer. Adaptive sampling projection decomposes the input image into multiple sub-images and samples a set of reconstruction points in each sub-image. Then, according to the position and color value of the sampling point, calculate the gray value of the reconstructed point and output the image. We use an adaptive sampling strategy to project the original feature into the feature interaction space to generate a projected feature.

#### Interaction graph reasoning

Interactive graphic reasoning is a graphical representation of multiple entities, concepts and their relationships. It can also be used for automatic reasoning and decision-making, helping people to better manage and control feature information. We put the projection feature into Interaction Graph Reasoning, defined g as graph adjacency matrix on k nodes, w as weight matrix, and the expression of graph convolution operation is as follows,

$$\begin{array}{c} X_{G}=\sigma (A_{G}X_{P}W_{G}), \end{array}$$ (1)

$$\begin{array}{c} =\sigma ((I-\hat{A^{G}})X_{P}W_{G}), \end{array}$$ (2)

Where *σ*() is the activation function of sigmoid. Firstly, Laplacian smoothing is applied, and the adjacency matrix is updated to $\left(I-\hat{A^{G}}\right)$, so that the node features are distributed throughout the graph. In practice, we use 11 convolution layer to implement $\hat{A^{G}}$ and *W*_*G*_.

#### Context guided attentive CRF fusion module

The method of CGA-CRF proposed by Liu et al. uses high-dimensional and discriminative features of context capture encoder stage in convolution space and feature interaction graph. The context-guided attention conditional random field is then used to selectively aggregate the features generated from different contexts and learn to generate the optimal features which are combined with the decoder to accurately segment tumors. To make the best use of the features learned by the encoder, we apply the CGA-CRF module to HDCNet, using the feature interaction graph to simulate and learn the relationship between lesion tissue and its surroundings, and selectively aggregate down-sampling features combined with skip connections to accurately locate brain tumors, segment tumor boundaries and improve boundary blur.

**Fig 4:**
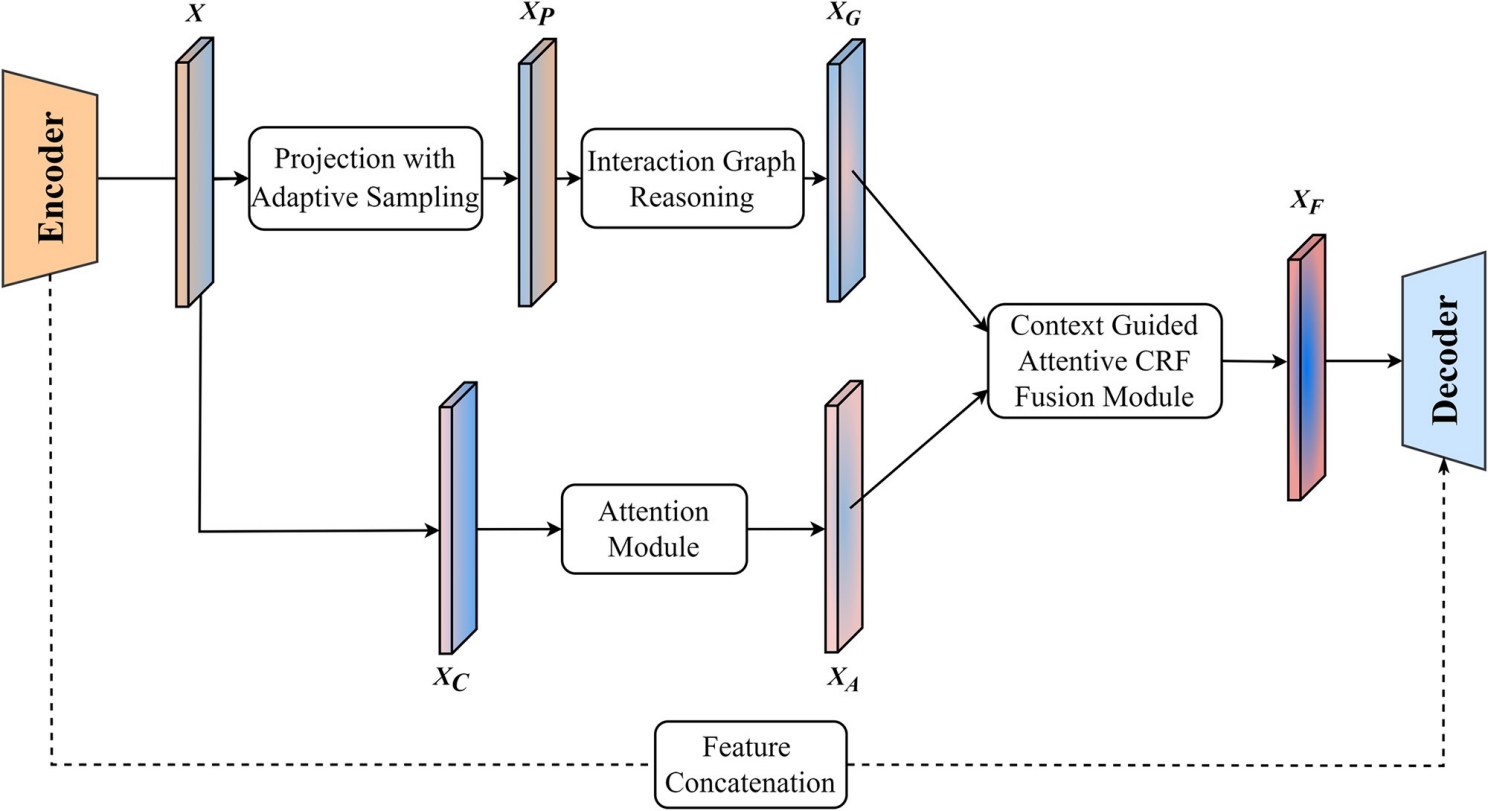
Feature interaction module.

As shown in Fig 4, we follow the feature interaction diagram module in CANet, and project feature *X* from the encoder using the projection of adaptive sampling, thus generating *X*_*P*_. Then the graph context information *X*_*G*_ is generated using the feature interaction graph to distinguish the tumor boundary. To make the network pay attention to the context information without losing the tumor information, we add a new attention module after the *X*_*C*_ generated by convolution. The attention mechanism enhances the interaction between dimensions while preserving channel and spatial information. Given a convolution context branch feature mapping *X*_*C*_, the attention module derives the attention map along the two independent dimensions of channel and space in turn, then multiplies the attention map by the input feature map for adaptive feature refinement, thus obtaining *X*_*A*_. Experiments show that the attention mechanism can induce the network to correctly focus on the tumor targets.

Research [42] shows that simply fusing features from different sources using channel-level connection or element-level summation mechanisms simplifies the relationship between feature maps from different sources and may lead to information loss. To make full use of the generated context information *X*_*G*_ and tumor features *X*_*A*_, we input *X*_*G*_ and *X*_*A*_ into the context-guided attention CRF fusion module with powerful reasoning ability. We can learn the hidden representation of the features encoded by the backbone of a neural network and then improve the generalization ability of the segmentation model. In addition, the potential features optimized by the conditional random field model can be learned, to realize the final feature fusion. CGA-CRF uses the context information *X*_*G*_ and the attention visual feature *X*_*A*_ to generate the final feature *X*_*F*_. To make the network retain the original low-level features, we skip Feature X from the encoder to the decoder to assist *X*_*F*_ in generating the best segmentation map related to the MRI image.

### Attention module

Visual attention mechanism is an innate ability of the human brain. Exploration of attention mechanisms aims to achieve selective attention to certain things while ignoring others in deep neural networks. In recent years, various attention mechanisms have been investigated to make models aware of the importance of different local information in images and to improve the overall performance of computer vision tasks. Convolutional block attention module (CBAM) [48] selectively designs two sub-modules of modal and spatial attention. Given an intermediate feature mapping as input, CBAM successively deduces the 1D modal attention mapping and 2D spatial attention mapping. GAM adopts the sequential channel spatial attention mechanism of CBAM and redesigns its sub-modules to improve the global attention performance of deep neural networks by reducing information diffusion and amplifying the global interactive representation.

To enhance the focus on the target tumor and retain information to amplify interactions across dimensions, we introduced the GAM, which includes 3D permutation with a multi-layer perceptron (MLP) for channel attention alongside a convolutional spatial attention sub-module. The channel attention sub-module uses latitudinal alignment to retain information in different dimensions and uses MLP to amplify cross-dimensional channel-space dependencies. The spatial attention submodule uses two convolutional layers for spatial information fusion, which makes the channel more aware of spatial information. Given the input feature mapping *F*_1_, the intermediate state *F*_2_ and output *F*_3_ are defined as follows,

$$\begin{array}{c} {\text{F}}_{2}={\text{M}}_{c}({\text{F}}_{1})⊗{\text{F}}_{1}, \end{array}$$ (3)

$$\begin{array}{c} {\text{F}}_{3}={\text{M}}_{s}({\text{F}}_{2})⊗{\text{F}}_{2}, \end{array}$$ (4)

**Fig 5:**
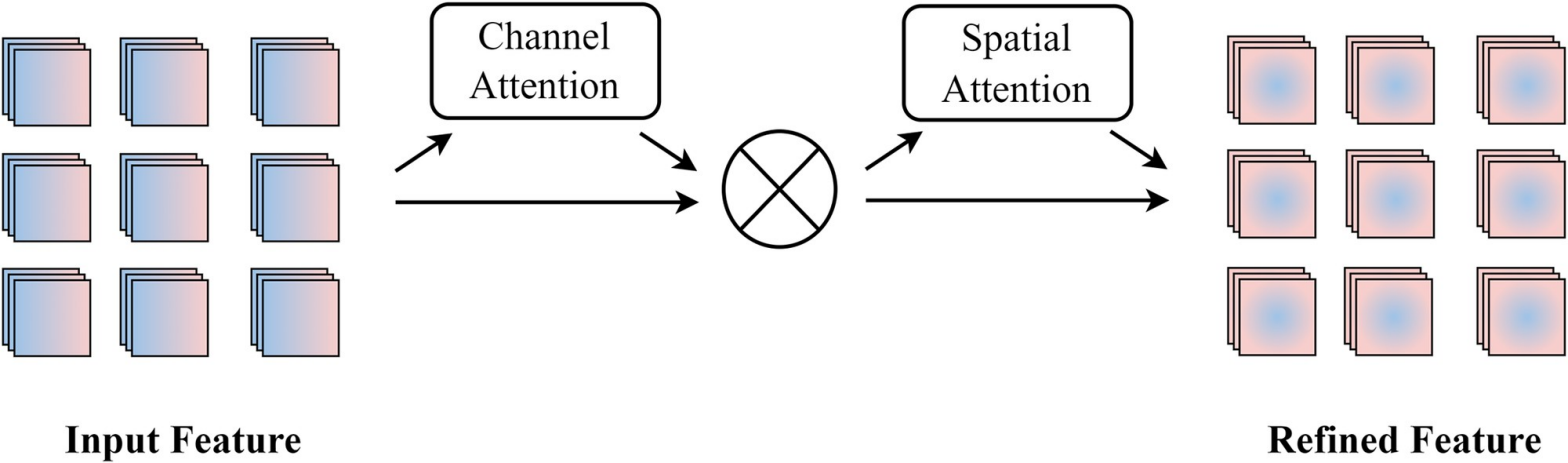
Global attention mechanism.

The whole calculation process is shown in Fig 5, where ⊗ denotes elemental multiplication; *M*_c_ and *M*_s_ are the channel and spatial attention maps, respectively.

### Network

As shown in the Fig 2, we introduce the interaction hierarchical decoupled convolution network with the classical encoder and decoder architecture. The feature interaction module consists of the CGA-CRF module and GAM module composition. The former is used to extract the context information between tumor boundaries and generate rich and consistent pixel-level features, while the latter introduces channel and spatial attention sub-module to locate the tumor and further enhance the feature representation ability.

The interaction hierarchical decoupled convolution network is a lightweight variant of 3D UNet, which has a symmetric encoder-decoder structure and a hop connection connecting the two paths. Similarly, we use the HDC module instead of 3D convolution to efficiently explore multi-scale and multi-view spatial environments. To alleviate the problem of label imbalance, we first cut the original image into 128 × 128 × 128 voxel space and use it as the input, and then use periodic down-shuffling (PDS) operation [49] before down-sampling. The purpose of PDS operation here is to rearrange a high-resolution input tensor *T*_original_ of size *C*_in_ into a low-resolution tensor *T*_output_ of size C, where H is the spatial size of *T*_original_ and *C*_in_ is the number of channels. The space size of *T*_output_ is half of the input space, and the output channel *C*′ is 8 × *C*. The specific operation of PDS is described as follows,

$$T_{\text{output}\;}(c^{\prime },x^{\prime },y^{\prime },z^{\prime })=T_{\text{original}\;}(\;\text{mod}\;(c^{\prime },C_{\text{in}\;}),$$ (5)

$$x^{\prime }\cdot 2+\lfloor \;\text{mod}\;(c^{\prime },2\cdot C_{\text{in}\;})/C_{\text{in}\;})\rfloor ,$$ (6)

$$y^{\prime }\cdot 2+\lfloor \;\text{mod}\;(c^{\prime },4\cdot C_{\text{in}\;})/(4\cdot C_{\text{in}\;}))\rfloor ,$$ (7)

$$z^{\prime }\cdot 2+\lfloor c^{\prime }/(4\cdot C_{\text{in}\;})\rfloor )),$$ (8)

where *c*′, *x*′, *y*′, *z*′ are the coordinates of the *T*_output_.

A three-dimensional convolution with a convolution kernel size of 3 × 3 × 3 and a step size of 1 is used in the first stage of the encoder, Rectified Linear Unit (ReLU) with a slope of 0.01, and synchronized normalization is applied after every convolution operation. In the feature coding stage, we use the HDC module in the last three coding units to convey multi-scale information, which benefits from the unique perception ability in layered decoupling convolutions. Similarly, in the decoding stage, in the middle two down-samples, we cascade the high-resolution features of the encoder with the features of the decoder. We replace the original skip connection with a more complex feature interaction module in the last down-sampling to make the network learn more accurate details. Trilinear interpolation is used for up-sampling in the last layer of the network, and then high-resolution segmentation results are output by softmax.

## Experimental results and analysis

### Datasets

The Multimodal Brain Tumor Segmentation (BraTS) Challenge is a global medical image segmentation challenge co-organized by the International Association for Medical Image Computing and Computer-Assisted Intervention (MICCAI) that focuses on automated segmentation algorithms for evaluating brain tumors. We evaluate the proposed method based on clinical data from BraTS 2018 and 2019 datasets. BraTS 2018 consists of 285 training sets and 66 validation sets, and BraTS 2019 consists of 335 training sets and 125 validation sets. The ground truth for all training cases is public; The ground truth for validating use cases is reserved for online evaluation. The ground truth image segmentation consists of five labels: background, gangrene and non-enhanced tumor, edema, enhanced tumor. Although a variety of different tumor labels are provided, they can be divided into three distinct tumor subregions in medicine for evaluation: whole tumor (WT), core tumor (CT), and enhanced tumor (ET). Each case contains the four different modalities described above (T1, T1ce, T2, Flair). The provided data were pre-processed by the organizers, including co-registration of the same anatomical template, interpolation of uniform isotropic resolution (1*mm*^3^), and skull dissection. All public data can be found at: https://ipp.cbica.upenn.edu/.

### Experiment details

We used PyTorch to implement the proposed method, and all experiments were carried out on two parallel Tesla T4 GPU. During training, we used the Adam algorithm to optimize the network. The batch size was 8, and the weight attenuation was 5 × 10^−4^. We set the initial learning rate to 1 × 10^−4^ decaying on a polynomial schedule. We adopted the Adam optimizer with an initial learning rate of *α* = 10^−3^. To take advantage of the spatial background information of the image, we used 3D images, which we cropped and scaled from 240 × 240 × 155 to 128 × 128 × 128. To expand the training data, we used the following data expansion techniques: (1) random mirror flip in the axial, coronal, and sagittal planes with a probability of 0.5; (2) random rotation between [−10°, 10°]; (3) random intensity shifted between [−0.1, 0.1] and the scale of between [0.9,1.1]. The L2 norm was used for model regularization with a weight decay rate of 10^−5^. During the testing phase, we zeroed the MRI data with the depth dimension of 240 × 240 × 155 to 240 × 240 × 160 and used it as the network input. How to solve the extremely uneven foreground and background areas in medical image segmentation is a major challenge, and it is extremely essential to select the appropriate loss function. The generalized Dice has been shown to be a good loss function to solve the imbalance of brain tumors, and its mathematical calculation formula is as follows,

$$\begin{array}{c} \text{GDL}=1-2\frac{\sum_{l=1}^{4}\omega_{l}\sum_{i=1}^{N}p_{ln}t_{ln}}{\sum_{l=1}^{4}\omega_{l}\sum_{i=1}^{N}p_{ln}+t_{ln}}, \end{array}$$ (9)

where *p*_*ln*_ represents the true pixel category of category *l* at the nth position, while *t*_*ln*_ represents the corresponding predicted probability value *wl* represents the weight of each class, that is $\omega_{l}=1/(\sum_{n=1}^{N}g_{ln}{)}^{2}$, where *N* represents all voxels, *l* represents the number of classes, *p* represents predicted voxels, and *t* represents true voxels.

### Evaluation metrics

We evaluate network performance using Dice similarity coefficient(%) and Hausdorff distance(95%) (HD95) as quantitative metrics. Dice calculation relies on the volume overlap between the predicted mask and the ground truth. Dice is sensitive to the internal padding of the mask, while HD95 is computed between boundaries of the prediction results and ground truth, which measures the segmentation accuracy of the boundary, defined as,

$$\text{Dice}=\frac{2\text{TP}}{\text{FN}+\text{FP}+2\text{TP}},$$ (10)

$$\text{HD95}(P,T)=\text{max}\{\underset{p\in \partial P_{1}}{\text{sup}}\;\underset{t\in \partial T_{1}}{\text{inf}}\;d\left(p,t\right),\;\underset{t\in \partial T_{1}}{\text{sup}}\;\underset{p\in \partial P_{1}}{\text{inf}}\;d\left(t,p\right)\},$$ (11)

Where TP, FP, and FN are true positive, false positive, and false negative respectively. For HD95, *P* represents the predicted value, *T* stands for the ground truth.

### Results

We validated the proposed method using the BraTS 2019 validation dataset and compared our method with the classical method. The performance comparison is shown in Tables 1 and 2. Because of the inherent characteristics of gliomas that make segmentation of ET and TC subregions more challenging compared to whole tumor segmentation, our proposed method scored 77.5%, 90.0%, and 82.7% for ET, WT, and TC, respectively. However, because our baseline model is a pseudo-3D model, there are still some gaps between our method and the best 3D methods, such as the BraTS2019 competition best method [16], but our model parameters are much less. On BraTS 2019 dataset, the Dice scores of ET, WT, and TC were 6.6%, 3%, and 5% higher than those of ResU-Net [50]; 3.8%, 0.6%, and 2% higher than those of 3D UNet [51]; and 1.4%, 1% and 4.8% higher than those of 3D FCN [52], respectively.

**Table 1:** Quantitative results of the proposed method on the Brats2019 validation set, including Dice and HD95.

Methods	Dice			HD95		
	ET	WT	TC	ET	WT	TC
Di Ieva et al. 2021 [62]	0.675	0.870	0.711	-	-	-
Zhang et al. 2020 [50]	0.709	0.870	0.777	-	-	-
Rehman et al. 2021 [58]	0.708	0.869	0.775	-	-	-
Jiang et al. 2019 [16]	0.802	0.908	0.863	3.21	4.44	5.86
Zhao et al. 2019 [63]	0.702	0.893	0.800	4.77	5.08	6.47
Wang et al. 2019 [51]	0.737	0.894	0.807	5.99	5.68	7.36
Li et al. 2019 [64]	0.771	0.886	0.813	6.03	6.23	7.41
Myronenko et al. 2019 [65]	0.800	0.894	0.834	3.92	5.89	6.56
Liu et al. 2021 [9]	0.759	0.885	0.851	4.81	7.09	8.41
Sun et al. 2020 [52]	0.761	0.890	0.779	-	-	-
Ali et al. 2021 [66]	0.740	0.880	0.810	6.47	8.52	7.45
Tong et al. 2022 [56]	0.751	0.885	0.776	-	-	-
**Proposed method**	0.775	0.900	0.827	4.17	4.62	6.59

“-” indicates that the number of parameters is not given in the original paper.

**Table 2:** Quantitative results of the proposed method on the Brats2018 validation set, including Dice and HD95.

Methods	Dice			HD95			Params
	ET	WT	TC	ET	WT	TC	
Nuechterlein et al. 2018 [53]	0.737	0.883	0.814	5.30	5.46	7.85	3.63M
Zhang et al. 2020 [50]	0.772	0.872	0.808	3.57	5.62	8.36	7.50M
Kao et al. 2018 [54]	0.787	0.905	0.813	3.81	4.32	7.56	9.45M
Chen et al. 2019 [35]	0.801	0.906	0.845	3.06	4.66	6.44	3.88M
Sun et al. 2020 [55]	0.772	0.892	0.763	5.04	5.48	10.62	20.31K
Tong et al. 2021 [56]	0.787	0.886	0.801	-	-	-	61.83K
Liu et al. 2021 [9]	0.767	0.898	0.834	3.86	6.89	7.67	3.34M
Sun et al. 2020 [52]	0.771	0.900	0.795	-	-	-	27.42M
Chandra et al. 2018 [57]	0.767	0.901	0.813	7.57	6.86	7.63	59.74M
Rehman et al. 2021 [58]	0.773	0.894	0.826	-	-	-	31.4M
Isensee et al. 2018 [59]	0.796	0.908	0.843	3.12	4.79	8.02	1.45M
Puch et al. 2018 [60]	0.758	0.895	0.774	4.50	10.66	7.10	1.48M
Carver et al. 2018 [61]	0.710	0.880	0.770	4.46	7.09	9.57	2.20M
Chen et al. 2018 [53]	0.749	0.894	0.831	4.43	4.72	7.75	3.32M
Myronenko et al. 2018 [17]	0.816	0.904	0.860	3.81	4.48	8.28	2.01M
**Proposed method**	0.800	0.902	0.841	2.36	3.94	6.88	1.21M

“-” indicates that the number of parameters is not given in the original paper. ET enhance tumor, WT whole tumor, TC tumor core.

**Fig 6:**
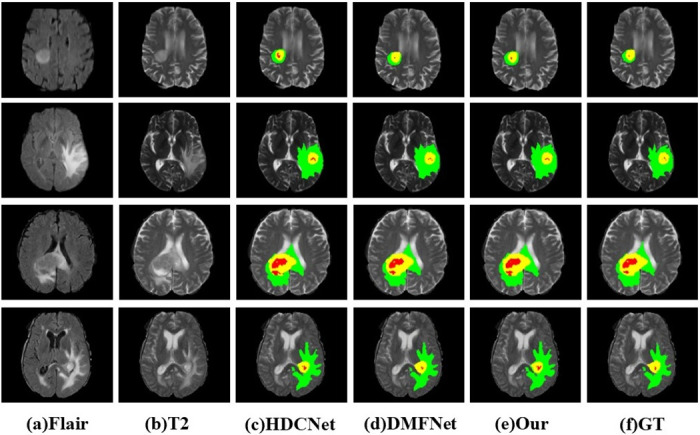
Segmentation results obtained by applying our proposed method to four cases on the BraTS2019 dataset. From left to right: (a,b) Flair and T2 slices, (c,f) 2D ground truth overlaid on T2 slices, ET: Yellow; TC: Yellow + Red; WT: Yellow + Red + Green.

Thus, our algorithm is more efficient and achieved comparable segmentation accuracy with fewer parameters. We also visually compared brain tumor segmentation results from various methods, including DMFNet, and HDCNet. Fig 6 shows our method. The feature interaction monomer approach allows the model to generate better segmentations (especially at class boundaries).

### Ablation study

Quantitative evaluation: The main contributions of this study are the addition of the feature interaction module to the encoder-decoder structure skip connection upon exploring the limitations of the UNet skip connection to achieve information interaction, and the introduction of a lightweight attention mechanism in the feature interaction structure to better learn the features of the tumor and improve the segmentation accuracy. To evaluate the validity of the model, corresponding ablation experiments were performed on the components of the model on the BraTS2019 dataset. In Table 3, with other settings such as network depth, parameter size, and training strategy unchanged, we verify the performance of the proposed method on a local validation set with/without feature interaction and attention mechanisms. The results show that the performance of the segmentation model on ET, WT and TC is improved by adding modules separately. Also, combining the two modules into the model further improves the segmentation performance on all metrics. Thus, compared to the original baseline model, our approach improves the Dice scores by 1.5%, 0.5%, and 0.7%, respectively, and reduces the Hausdorff distances for the segmentation of ET, WT, and TC by 0.15, 3.05, and 0.90, respectively.

**Table 3:** Ablation study of the proposed method on the Brats2019 validation dataset with/without the feature interaction module and the attention module. Performance is measured in Dice (%) and Hausdorff distance (*mm*).

Methods	CGA	GAM	Dice			HD95		
			ET	WT	TC	ET	WT	TC
Baseline			0.760	0.895	0.820	4.32	7.67	7.49
**Proposed method**	✓		0.772	0.899	0.818	2.85	4.75	6.31
**Proposed method**		✓	0.777	0.902	0.819	2.71	4.65	6.00
**Proposed method**	✓	✓	0.775	0.900	0.827	4.17	4.62	6.59

**Fig 7:**
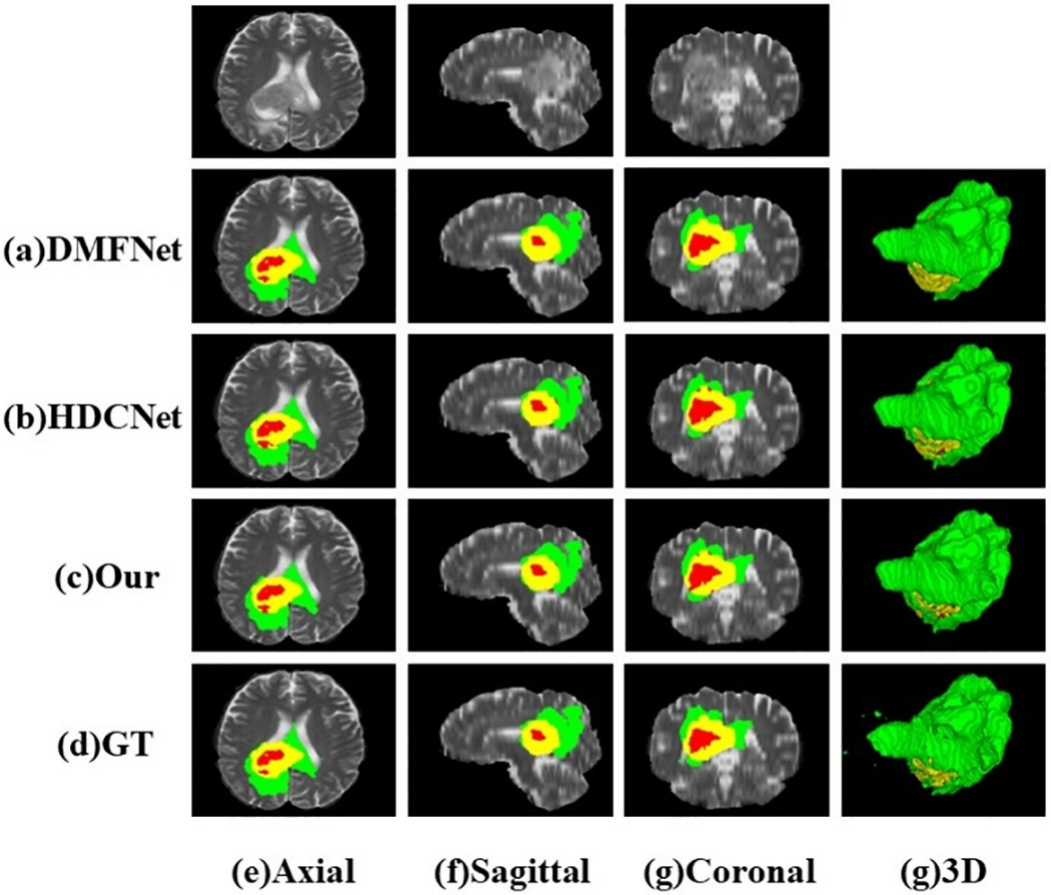
Visualisation results superimposed on T2 slices at different latitudes, the horizontal axis represents the different latitudes, the last column shows the 3D visualisation results, the vertical axis represents the results of the different methods and the last row shows the ground truth mask.

**Fig 8:**
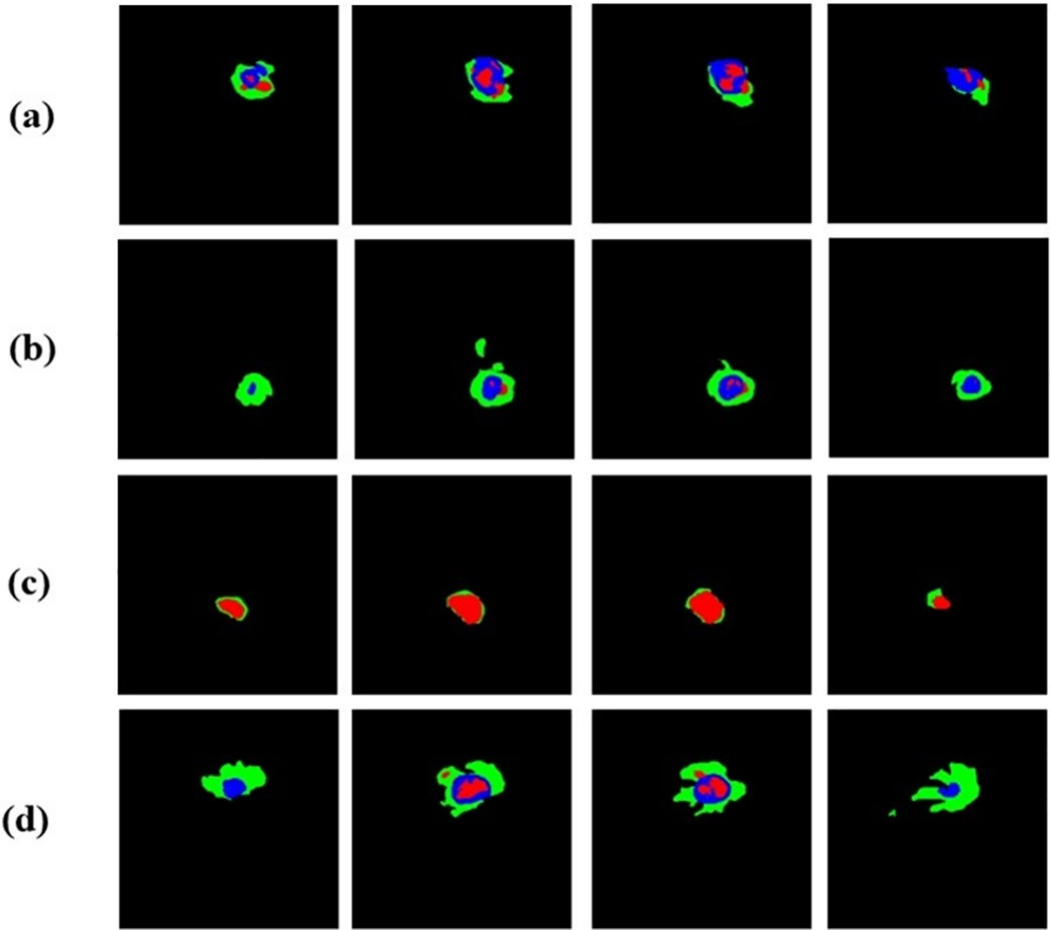
Samples of Multimodal Brain Tumor Segmentation on BraTS 2019, from left to right, are the segmentation feature maps generated after the addition of the attention mechanism, and we have selected the feature slices from different periods for easier observation, where green represents WT, blue represents ET, and red represents TC.

Qualitative comparison: Because the ground truth labels of the BraTS validation set were not publicly available, a random selection of cases from the training set was used to form a local validation set to facilitate quantitative evaluation. The segmentation results and the 3D visualization are shown in Fig 6. Compared with baseline network and DMFNet, the results generated by our method are closer to the basic facts, especially in boundary segmentation, and our method realizes better tumor boundaries. In Fig 7, we show the segmentation results of different imaging angles, and the last column is 3 d segmentation visualization. Both the quantitative evaluation in Table 3 and the qualitative comparison in Fig 7 demonstrate the reliability and effectiveness of our proposed method. We also visualized the feature map of the proposed method. As can be seen in Fig 8, the feature maps generated by adding the attention module focus more on the target region, which facilitates segmentation. In addition, in order to further prove the advantages of this model, we made a detailed analysis of the model parameters, as shown in Table 4. Compared with the traditional 3D-UNet parameters, this method has less segmentation and higher segmentation accuracy. Although the efficiency of this model is slightly lower than that of the larger model NVDLMED [17], our parameters are much smaller.

**Table 4:** Compare our method with the methods commonly used on the BraTS 2018 dataset.

Method	Dice	FLOPs(G)	Params(M)
	WT		
3D-UNet [29]	0.885	1669.53	16.21
NVDLMED [17]	0.907	1495.53	40.06
Baseline	0.897	25.53	0.29
Baseline+CGA-CRF	0.899	26.26	0.47
Our	0.902	28.46	1.20

## Discussion and conclusion

Segmentation of brain tumors plays an important role in diagnosis, treatment planning and evaluation of brain tumors. In this study, a comprehensive approach was adopted to integrate the characteristics of encoder learning in order to obtain more accurate semantic information and further enhance the network’s ability to accurately locate and segment tumors.

Compared with traditional methods, our method has the following advantages: First, we use HDC module to reduce the requirement of GPU memory in training. Second, we replace the traditional skip connection structure to realize mutual learning among features. Third, the feature interaction module is introduced into U-shaped network to realize segmentation of brain tumor regions with blurred contour. Finally, this paper introduces the attention mechanism, so that the complementary features in different patterns can be learned, and the network can focus on the most useful features.

Due to the challenge of medical image segmentation, the segmentation results of our method is unstable compared with the large model, and complex environmental factors need to be considered in practical application, so a lot of experiments are needed to verify its practicability. In addition, the segmentation efficiency of the network is also important for future practice. In future work, we will make use of the characteristics of multimodal data and fuse different patterns to develop a more effective and accurate segmentation model, and expand the application of this method to verify the application of our method in various other types of segmentation task.

## Supporting information

S1 File (DOCX)
