# Supplementary material for: Feature interaction network based on hierarchical decoupled convolution for 3D medical image segmentation
Source: PLoS One. 2023 Jul 13;18(7):e0288658. doi: 10.1371/journal.pone.0288658 (PMC10343050; doi:10.1371/journal.pone.0288658)
Supplement: S1 File — (DOCX) [file pone.0288658.s001.docx]

**Dataset**

Dear Editor, thank you for pointing out the shortcomings in our paper, especially in the data information section. In this paper, we used the BraTS 2018 and BraTS 2019 datasets issued by the Center for Biomedical Image Computing and Analytics. Since data set acquisition requires registration and application, we promise not to upload data set to other public sites, therefore due to access problems, we can't upload the complete data sets, but all the data are free, open, they can be found at <https://ipp.cbica.upenn.edu/>. More detailed information can be found at <https://www.med.upenn.edu/cbica/>.

In addition, we uploaded some of our segmentation results, which you can find at DOI: **10.6084/m9.figshare.23542575** as proof of the authenticity of our experimental data.
